# Extended Spectrum β-Lactamase (ESBL) Producing *Escherichia coli* in Pigs and Pork Meat in the European Union

**DOI:** 10.3390/antibiotics9100678

**Published:** 2020-10-07

**Authors:** Ieva Bergšpica, Georgia Kaprou, Elena A. Alexa, Miguel Prieto, Avelino Alvarez-Ordóñez

**Affiliations:** 1 Department of Food Hygiene and Technology, Universidad de León, 24007 León, Spain; gdkaprou@gmail.com (G.K.); ealexandra.alexa@gmail.com (E.A.A.); miguel.prieto@unileon.es (M.P.); 2 Institute of Food Safety, Animal Health and Environment BIOR, LV-1076 Riga, Latvia; 3 Institute of Food Science and Technology, Universidad de León, 24007 León, Spain

**Keywords:** ESBL, *E. coli*, prevalence, pigs, pork meat, EU

## Abstract

The aim of this article is to review the fast and worldwide distribution of ESBL enzymes and to describe the role of the pork production chain as a reservoir and transmission route of ESBL-producing *Escherichia coli* and ESBLs in the European Union (EU). The use of β-lactam antibiotics in swine production and the prevalence of ESBL producing *E. coli* in fattening pigs and pork meat across Europe is analyzed. Overall, an increasing trend in the prevalence of presumptive ESBL producing *E. coli* in fattening pigs in the EU has been observed in the last decade, although with major differences among countries, linked to different approaches in the use of antimicrobials in pork production within the EU. Moreover, the various dissemination pathways of these bacteria along the pork production chain are described, along with factors at farm and slaughterhouse level influencing the risk of introducing or spreading ESBL producing bacteria throughout the food chain.

## 1. Introduction

Antimicrobial resistance (AMR) is a global concern in public health, threatening to complicate the treatment of infections worldwide. Emergence and spread of AMR has been attributed to the misuse or overuse of antibiotics in human and veterinary medicine. Antimicrobial resistance occurs as microorganisms modify their genetic information when they are exposed to antimicrobial drugs [1].

The use of antibiotics dates back to the beginning of the 20th century, when Paul Ehrlich discovered a chemical called arsphenamine which was effective against *Treponema pallidum*, the bacterium causing syphilis [2]. Twenty years later, in 1928, Alexander Fleming isolated penicillin, a β-lactam antibiotic from the fungus *Penicillium notatum* and, since then, the strike of the antibiotic’s era began.

β-lactams, such as penicillins, carbapenems, monobactams, and cephalosporins account for 60% (by weight) of all antibiotics used worldwide, and in human medicine are one of the most widely prescribed antibiotic classes [3,4]. Intensive use and misuse of β-lactam antibiotics both in human and in veterinary medicine has led to the spread of extended spectrum β-lactamase (ESBL) producing resistant bacteria. The World Health Organization (WHO) has indicated that third generation resistant *Enterobacteriaceae*, including ESBL-producing *Enterobacteriaceae*, are among the world’s most serious and critical threats of the 21st century [5].

**Figure 1:**
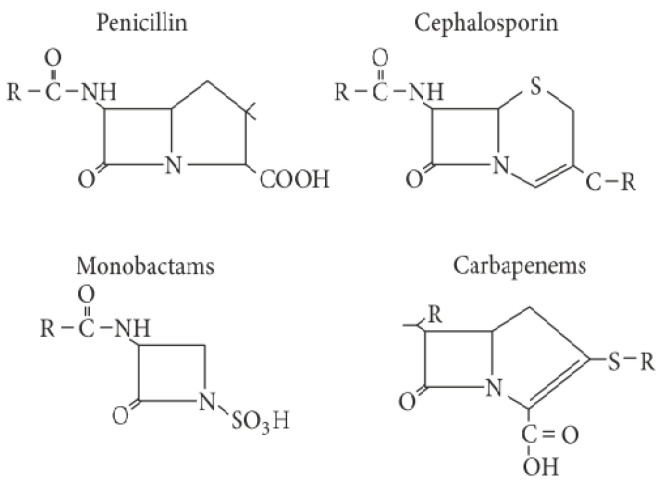
Structure of β-lactam antibiotics, adapted from [12].

β-lactams interfere with the synthesis of the bacterial cell wall, which results in the inhibition of bacterial growth, by binding to penicillin-binding-proteins (PBPs) that are enzymes involved in the synthesis of peptidoglycan. β-lactam antibiotics are used to treat infections caused by both Gram-positive and Gram-negative bacteria. They all share as a common structure a four-membered lactam ring, known as the β-lactam ring (Figure 1). This is a cyclic amide with a heteroatomic ring structure that consists of three carbon atoms and one nitrogen atom [6]. β-lactam antibiotics act on the formation of the bacterial cell wall by interfering with the PBPs at the final stage of peptidoglycan synthesis [6].

The intensive use of β-lactam antibiotics for the past 70 years has led to the evolution of β-lactam resistance in bacteria. Resistance against β-lactam antibiotics in bacteria can be ensured through three different mechanisms. The first mechanism includes the mutation in genes encoding for PBPs, the creation of mosaic PBPs or obtaining alternative PBPs [6]. The second mechanism consists of changes in the permeability of the cell wall that could be due to alterations in the expression of porins or active efflux pumps [6]. However, the most frequent mechanism is the third one—the inactivation of the antibiotic by the expression of β-lactamases [6].

The first β-lactamases were discovered in 1940 by Edward Penley Abraham [7]. β-lactamases are enzymes produced by bacteria that inactivate the β-lactam ring by breaking the amide bond of the β-lactam ring and adding a water molecule to the ring-opened molecule. Narrow spectrum β-lactamases are also called penicillinases or cephalosporinases, depending on the target. Moreover, some Gram-negative bacteria, especially members of the *Enterobacteriaceae* family, and Gram-positive bacteria, e.g., *Staphylococcus aureus*, can also produce an array of extended spectrum β-lactamases (ESBLs), enzymes that hydrolyze many different β-lactams and can cause resistance to oxyimino-cephalosporins (cefotaxime, ceftazidime, ceftriaxone, cefuroxime, cefepime) and monobactams (aztreonam), but not to cephamycins (cefoxitin, cefotetan) or carbapenems (imipenem, meropenem, ertapenem, doripenem) [8,9,10]. ESBLs are inhibited by ESBL inhibitors, such as clavulanate, sulbactam and tazobactam (older β-lactamase inhibitors) and avibactam, relebactam and vaborbactam (latest Food and Drug Administration (FDA)-approved inhibitors), which are therefore frequently included with β-lactam antibiotics in the formulation of therapeutic drugs [11].

As β-lactamases share some sequence homology with PBPs, it is considered that they have evolved from them [10,13]. β-lactamase encoding genes (*bla*) can be located in chromosomes or plasmids. The first plasmid-mediated β-lactamase (TEM-1) in Gram-negatives was described in 1965, from an isolate from a patient named Temoniera in Greece, therefore named TEM [14]. Since then, hundreds of different β-lactamases have been discovered, and the first ESBL (SHV-2) was discovered in Germany, isolated from a *Klebsiella ozonae* strain in 1985 [15]. The CTX-1 enzyme was discovered in 1985 from *Klebsiella pneumoniae*, isolated from patients in an intensive care unit in France [16]. Nowadays, it is the most widely spread β-lactamase in food-producing animals [17].

According to the reports of the European Food Safety Authority (EFSA) the prevalence of presumptive ESBL producing *E. coli* in fattening pigs and pork meat varies a lot within the EU countries [18,19]. It is worth noting that there are several steps within the pork production chain where pigs are exposed to ESBL producing bacteria and can become carriers of them, such as trading places, where new animals are mixed with older animals within the same herd, or slaughterhouse waiting areas [20]. In addition, cross-contamination in slaughterhouses, especially at evisceration, poses a risk of carcass contamination with ESBL producing *Enterobacteriaceae* [21,22]. This review article is aimed at discussing and analyzing aspects related to the occurrence and transmission routes of ESBL producing *Enterobacteriaceae* in the pork production chain.

## 2. Classification of ESBLs

The number of ESBLs reported is constantly growing [12]. They are subdivided into ten families based on their amino acid sequences—CTX-M, TEM, SHV, SFO, PER, VEB, GES, TLA, BES and OXA. In this review article the focus will be on CTX-M, TEM and SHV ESBLs as they are the most common among members of the *Enterobacteriaceae* family. There are two main characterization schemes of β-lactamases—the Ambler’s scheme and the Bush–Jacoby–Medeiros scheme [23,24]. The Bush–Jacoby–Medeiros classification, which is based on functional similarities among β-lactamases (i.e., substrate and inhibitor profile), recognizes a total of 11 groups to classify β-lactamases. Extended-spectrum β-lactamases are assigned to group 2be if the hydrolysis rates for ceftazidime, cefotaxime or aztreonam are by 10% higher than for benzylpenicillin [24]. According to the Ambler’s classification, β-lactamases are subdivided in four classes (A–D) [23]. These classes differ in their mechanisms of action and active sites. Class A, C, and D β-lactamases have serine at their active sites and undertake hydrolysis of serine esters, while class B β-lactamases have zinc ion(s) at their active site, catalyzing the hydrolysis of almost all β-lactam antibiotics, with the exception of monobactams [25,26]. There are more than 500 enzymes that belong to the class A of β-lactamases, including the ESBL variant TEM, CTX-M, and SHV enzymes (Table 1).

Apart from TEM, CTX-M and SHV ESBLs, there are other ESBLs which are not so common among members of the family *Enterobacteriaceae* and will not be covered in this review article, like PER-1 β-lactamases, which confer resistance to ceftazidime in *Acinetobacter baumanii* and *Pseudomonas aeruginosa* [28]; FEC and SFO ESBLs, which hydrolyze cefotaxime [29,30]; GES, CME and PER ESBLs, which hydrolyze ceftazidime [31,32,33]; BES and TLA β-lactamases, which can hydrolyze several substrates, like cefotaxime, ceftazidime and aztreonam [34,35], and OXA β-lactamases, which hydrolyze oxacillin and cloxacillin and are poorly inhibited by clavulanic acid. Even though most of the OXA-type β-lactamases do not hydrolyze extended spectrum cephalosporins, there are some exceptions. In 1991, the first ESBL-type OXA (OXA-11) enzyme, deriving from OXA-10, was recovered from a *P. aeruginosa* isolate which showed resistance to ceftazidime. Apart from the OXA-11 enzyme, several other ESBLs have derived from OXA-10 as well, such as OXA-13, OXA-14 [36], OXA-16 [37], OXA-17 [38], OXA-19 [39] and OXA-28 [40]. The OXA-15 enzyme, which derives from OXA-2 β-lactamases, also confers an ESBL phenotype [41]. ESBL OXA-type enzymes are mostly found in *P. aeruginosa* and have hardly been identified in other species, which indicates a probable low transfer rate between species [42].

Two main strategies for the evolution of ESBLs have been identified in *Enterobacteriaceae*: (i) the selection of mutants that have a wider spectrum of substrate hydrolysis and are derived from plasmid-mediated TEM and SHV type β-lactamases; and (ii) the acquisition from the environment of novel β-lactamase genes that encode ESBL enzymes [43].

TEM-type β-lactamases are responsible for ampicillin resistance among *Enterobacteriaceae* [44]. TEM-1 β-lactamases are plasmid- and transposon-mediated. This location has facilitated their spread to other bacterial species worldwide. They can be found in different members of the family *Enterobacteriaceae*, and in *P. aeruginosa*, *Haemophilus influenzae* and *Neisseria gonorrheae* [10]. Several hundred variants of TEM β-lactamases have been described and all of them have derived from TEM-1 and TEM-2 β-lactamases [45]. Single nucleotide polymorphisms (SNPs) in *bla*_TEM_ genes, which encode TEM β-lactamases, may lead to amino acid substitutions in the enzyme. The most common substitutions are those at the Glu^104^, Arg^164^, Glu^238^ and Glu^240^ positions [46]. The majority of TEM β-lactamases are ESBLs. Some of the TEM derivates have reduced affinity for β-lactamase inhibitors and are called inhibitor-resistant TEM. They also have negligible activity against extended-spectrum cephalosporins, and therefore they are not considered as ESBLs [6].

The probable ancestor of the enzyme SHV (sulfhydryl reagent variable) is a chromosomal penicillinase of *Klebsiella pneumoniae*. The first ESBL SHV enzyme was isolated from *Klebsiella ozaenae* in 1983, and, since then, various SHV types responsible for resistance to third generation cephalosporins have been described [15]. SHV β-lactamases can be subdivided into three subgroups based on their molecular characteristics or functional properties. Members of the subgroup 2b hydrolyze penicillins and early generation cephalosporins, and are inhibited by clavulanic acid and tazobactam; members of the subgroup 2br are broad-spectrum β-lactamases that are resistant to clavulanic acid; and members of the subgroup 2be hydrolyze one or more oxyimino β-lactams (ceftazidime, cefotaxime, and aztreonam) [47]. There are over one hundred allelic variants of SHV β-lactamases described [45]. Many of those are associated with resistance to third generation cephalosporins as well as monobactam and carbapenems [48].

Broad-spectrum SHV-1 β-lactamases dominate in *K. pneumoniae* and are responsible for up to 20% of plasmid-mediated ampicillin resistance in this species [49]. SHV enzymes can be found in different species, such as *Enterobacter cloacae*, *Salmonella*, *K. ozaenae*, *Citrobacter freundii* but more commonly in *K. pneumoniae* and *E. coli.* They are usually chromosomally encoded in *K*. *pneumoniae*, and plasmid-encoded in *E*. *coli* [10]. Some variants of SHV β-lactamases (e.g., *bla*_SHV-27_) have been found on plasmids simultaneously carrying several ESBL coding genes [47].

The active site of SHV β-lactamases is located within a cleft made by subdomains and contains a Ser^70^ residue that mediates the nucleophilic attack on the carbonyl group of the β-lactams ring [47]. ESBL SHV enzymes, compared to SHV-1, have one to six amino acid substitutions, what means that even single amino acid changes can lead to an ESBL phenotype. The most common substitutions are at Leu^35^, Gly^238^ and Glu^240^ positions [47]. Plasmid-borne SHV enzymes are commonly found in bacteria isolated from food producing animals and meat products [47].

The *bla*_SHV-1_ gene was first identified in 1970s in *E. coli* [50]. Until now, there are over 180 SHV allelic variants characterized. At least 46 of those are ESBL SHV encoding *bla*_SHV_ genes, and most of them have been detected in *E. coli* or *K. pneumoniae* [47]. Probably, SHV ESBLs have evolved from *bla*_SHV-11_ and *bla*_SHV-1_ genes [47]. SHV ESBLs have been described worldwide, most of them are associated with plasmids and have been reported as unique cases.

CTX-M-type β-lactamases hydrolyze cefotaxime, and there are over 220 different CTX-M-type enzymes characterised so far. CTX-M β-lactamases are widely disseminated within *Enterobacteriaceae*, e.g., *E. coli, S. enterica, C. freundii, Proteus mirabilis, E. cloacae, K. pneumoniae, Morganella morganii, Providencia* spp*.,* etc. [51,52,53]. However, CTX-M-type ESBLs have also been identified in some species apart from *Enterobacteriaceae,* such as *Pseudomonas aeruginosa, Acinetobacter* spp.,* Aeromonas* spp*., Stenotrophomonas maltophillia, Vibrio fluvialis and Vibrio cholerae*, indicating a high transmission rate of CTX-M-type genes [43]. *bla*_CTX-M_ genes found in clinical isolates of *Enterobacteriaceae* are mainly carried by conjugative plasmids, although in some species *bla*_CTX-M_ genes are integrated into chromosomes [43,54]. Olson and colleagues discovered in 2005 that the ancestor of the CTX-M-9 group of ESBLs is a chromosomal β-lactamase from *K. georgiana* [55]. CTX-M β-lactamases hydrolyze cefotaxime and ceftriaxone better than ceftazidime. However, the spectrum of hydrolysis varies by the enzyme, and there are CTX enzymes (CTX-M-15, CTXM-16 and CTX-M19) that exhibit enhanced catalytic efficiencies against ceftazidime [11,56].

CTX-M-type enzymes are susceptible to β-lactamase inhibitors (e.g., tazobactam and clavulanate) as well as to the new non β-lactam-derived β-lactamase inhibitor avibactam [57]. Since the first sporadic cases of CTX-M producing bacteria in the 1980s in Europe [58], Japan [29] and South America [59], the CTX-M-type ESBLs have outnumbered other ESBLs, such as TEM and SHV [43] and have become the most common type of ESBL [4]. The rapid spread of CTX-M- type ESBLs depends on many factors, such as the effective capture and dissemination of *bla*_CTX-M_ genes on mobile genetic elements (MGEs), the association of MGEs with highly successful bacterial clones, the low fitness cost imposed by CTX-M production, and the intensive use of expanded-spectrum cephalosporins and fluoroquinolones (which can co-select CTX-M-producing strains that are often resistant also to these drugs) in veterinary and clinical fields [43].

In 1990s the most frequently found ESBL genes were *bla*_TEM_ and *bla*_SHV_, but nowadays the most prevalent genes in livestock are *bla*_CTX-M_ [17,60]. There are hundreds of variants of CTX-M genes identified and they show higher activity against cefotaxime than against other oxyimino-β-lactams [9].

## 3. Dissemination of ESBLs

### 3.1. Dissemination Pathways of Antibiotic Resistance Genes

Many of the β-lactamase encoding resistance genes are believed to be originated from non-pathogenic bacteria [61]. Some bacteria are natural hosts of β-lactamases, such as *Streptomyces*, *Nocardia*, and *Actinomadura* [27]. However, other bacteria, including *E. coli,* can gain resistance genes via horizontal gene transfer (HGT) between bacteria belonging to the same or even different species [61,62]. Transfer of *bla* encoding genes can occur through different pathways, for example, vertical proliferation of bacteria or HGT via MGEs, that include transposons, integrons, insertion sequences (IS), plasmids or bacteriophages.

HGT can be carried out via transformation, conjugation or transduction. Transformation is a process where competent cells uptake plasmid DNA or chromosomal DNA fragments that have been released to the extracellular environment after the cell death [63]. Conjugation is the transfer of DNA through direct contact via cell surface pili or adhesins [61]. However, transduction requires additional organisms to transmit the genetic information. Indeed, in transduction, bacteriophages replicate within the donor organism and, at DNA packaging, sometimes incorporate DNA sequences from the host cell into a new host [61].

Plasmids can be mobilized in the same or different species, via conjugation. One bacterium can carry several plasmids encoding different antibiotic resistance genes (ARGs), depending on plasmid incompatibility (Inc). Incompatibility is a manifestation of the relatedness of plasmids. Therefore, compatible plasmids can survive together in transconjugants, but plasmids that are related will not be stably propagated together [54]. The *bla*_CTX-M_ gene is often associated in IncF group plasmids with other ESBL coding genes, e.g., *bla*_TEM-1_ and *bla*_OXA-1_ [54]. The dissemination of *bla*_CTX-M_ within members of the *Enterobacteriaceae* has been rapid due to its capability to be transferred by all major Inc plasmids, e.g., *bla*_CTX-M14_ is frequently associated with IncK plasmids [43,64], and it has been shown that Incl1 ST3 plasmids are a frequent vector for the dissemination of CTX-M-1 β-lactamases within food products of animal origin [17]. It has also been demonstrated that the ISEcp1 element and conjugative plasmids have facilitated the transfer of CTX-M genes to different species [65]. More information on the most prevalent and studied plasmids in the *Enterobacteriaceae* family can be found in Carattoli et al. [54].

Transposons can transfer resistance genes between plasmids, or into and out of the chromosome. *bla*_TEM_ genes are carried by three transposons, Tn*1*, Tn*2* and Tn*3*, respectively. All these transposons have transposase and resolvase genes, *tnp*A and *tnpR*, and a *res* resolution site [6]. Similarly, as *bla*_SHV_ originated from a *K. pneumoniae* chromosome, fragments of this chromosome together with the *bla*_SHV_ gene have been carried to plasmids by IS*26*-dependent mobilization.

Insertion sequences (ISs) are transposable elements responsible for the mobilization and transfer of ARGs. Some ISs, such as IS*CR1,* take part in the transmission of β-lactamase coding genes, ESBLs included [66]. Indeed, ISE*cp1B* and IS*CR1* play a major role in the transmission of the *bla*_CTX-M_ gene, because they can mobilize flanking DNA segments, and were involved in the capture of CTX-M genes from the *Kluyvera* spp. chromosome and their transposition to plasmids [66,67]. Also phage-related elements take part in the dissemination of the *bla*_CTX-M_ gene within members of the Familiy *Enterobacteriaceae* [68].

An integron is a genetic unit able to capture, mobilize and express genes that are contained in genetic elements known as gene cassettes [69]. Multidrug resistance is strongly associated with the presence of integrons, as they commonly carry a large array of resistance gene cassettes [70,71]. Yuan and colleagues highlighted that highly active ARG transfer might occur via class 1 integrons between *E. coli* and other bacteria, while *Poirel* et al. [72] showed that the mobilization of *bla*_CTX-M_ genes is associated with class I integrons [73].

### 3.2. Dissemination of ESBL-Producing E. coli in the Pork Production Chain

*E. coli* is a component of the normal human and animal gut microbiota. This feature makes *E. coli* one of the most probable vectors for the spread of ESBLs [74]. As explained in previous sections, the wide spread of ESBLs is ensured by plasmids and other MGEs [54].

An interesting example of transfer from food-producing animals to the human gut microbiome is *Escherichia coli* ST131, which is an extraintestinal pathogen which can colonize the gastrointestinal tract of food-producing animals and humans [75]. This sequence type of *E. coli* was believed to be selected in poultry during the 1940s and, since then, has been recognized as a vehicle for human exposure and infection [76]. Interestingly, it often carries different plasmids (sometimes also encoding ARGs) and has contributed to the dissemination of CTX-M β-lactamase encoding genes, and, less frequently, TEM, SHV and cephalomycinase (CMY) encoding genes [77].

High correlations exist between resistance profiles of *E. coli* isolated from food animals, like poultry and pigs, and those of isolates from humans with blood stream infections [78]. In addition, it has been shown that genetically distinct *E. coli* isolates from humans and animals carry nearly identical IncI1 plasmids that encode third-generation cephalosporin resistance determinants and probably contribute to the spread of ESBLs through food animals (e.g., pork, chicken) to humans [79].

Some of the initial phases where pigs can be colonized by ESBL-producing *E. coli* is at trading places, livestock transport vehicles, through introduction of new animals into herds, or at lairage in the slaughterhouse [20]. As drinking water [80], surface water [81] and wastewater [82], all can be contaminated with ESBL-producing *E. coli*, a possible transmission of ESBLs from the environment to pigs can occur. Holding pens in stables and at lairage in abattoirs are recognized as major hotspots for the transmission of ESBL producing *Enterobacteriaceae* along the pig production chain [83].

At slaughterhouses, a risk of cross-contamination of meat exists, especially during evisceration, where carcasses can be contaminated by AMR bacteria from the feacal content of the same or different pigs [21,22]. Food processing environments are considered to be important intermediate reservoirs and vectors of AMR bacteria, and also food handlers pose a risk of transmission of ESBL producing bacteria [84,85].

By performing whole-genome sequencing (WGS) to ESBL producing *E. coli* isolates obtained from farmers, poultry and pigs, an association was made between the isolates from farmers and pigs, as they showed only 1.8 single nucleotide polymorphism (SNP) mismatches per 1 Mb, but no association was established between isolates from humans and poultry as the SNPs per 1 Mb were in 1263 positions [79]. These data suggested that transfer of ESBL-producing *E. coli* clones between pigs and piggery workers occurs.

## 4. Use of β-Lactam Antibiotics in Pig Production

### 4.1. Usage of β-lactam Antibiotics and Emergence of Related AMR Determinants in Pig Production

There are three usages of antibiotics in swine industries: (i) as growth promoters, (ii) as prophylactic or metaphylactic treatments, to prevent disease, and (iii) as therapeutics for the treatment of acute infections. Antimicrobial agents were introduced to treat diseases in food-producing animals in 1940s, and in 1950s feed supplemented with antimicrobials was already used for cattle, pigs and poultry [86]. Prophylactic (individual animal) and metaphylactic (whole herd) treatments also work by adding the antibiotic to the animal feed. However, the supplemented feed is used for a few days and not on a regular basis. The concentration of antibiotics is higher than in growth promotion uses and can reach therapeutic concentrations [87]. For therapeutic purposes there are a wide range of antibiotics available, and they can be used either orally or by injection. Antimicrobials used in clinical therapy are of broad spectrum and are mainly active against Gram-negative bacteria, like *Salmonella* or *E. coli* [86].

The use of growth promoters was banned by the EU in 2006, according to the EC Regulation No. 1831/2003 [88], but some of them were taken out of the market even before. Sweden was the first country in the world to ban the use of antibiotics in animal feed since 1986. However, antibiotics are still in use as growth promoters in some countries outside the EU. China has not yet prohibited the use of antibiotics as growth promoters, however they are proposing restrictions [89,90], similar to the FDA in the USA.

In veterinary medicine, three groups of β-lactam antimicrobial agents are used, including penicillins, first- to fourth-generation cephalosporins and the β-lactamase inhibitors [6]. Regarding the penicillin type antibiotics, such as ampicillin, amoxicillin, benzylpenicillin, cloxacillin and hetacillin, they are used to treat infections caused by Gram-positive bacteria. They are commonly combined with β-lactamase inhibitors (e.g., amoxicillin-clavulanate) to prevent their degradation and inactivation [6]. First-generation cephalosporin antibiotics (cefadroxil, cefapirin and cephalexin) are administered as an alternative to treat staphylococcal and streptococcal infections. Second-generation cephalosporins (cefaclor, cefamandole, cefonicid, ceforanide and cefuroxime) have greater spectrum of activity against Gram-negative bacteria, while retaining some activity against Gram-positive bacteria. Cefovecin, cefpodoxime and ceftiofur, which are third generation cephalosporins, exhibit broad spectrum of activity and have increased activity against Gram-negative bacteria. Finally, fourth generation cephalosporins, such as cefquinome, which is used in veterinary medicine, have the broadest activity against Gram-negative as well as Gram-positive bacteria [6].

It has been shown that the use of specific antimicrobials, including third-generation cephalosporins (cefotaxime), strongly correlates with the level of resistance towards these antibiotics in commensal *E. coli* isolates from pigs [91,92]. Antibiotics used for growth promotion were initially from the same chemical families as the antibiotics used to treat human infections. They were added to the feed in low concentrations and for the whole life of the animal. This created the perfect environment for the selection of antibiotic resistant bacteria and the spread of ARGs among enteric bacteria in the pig gastrointestinal tract [87].

### 4.2. Sales of Penicillins and Cephalosporins in the EU

Based on FDA reports, Done and colleagues calculated that in the USA in 2011 around 80% of the antibiotics sold by weight were for animal usage [93].

The sales of penicillins, and first to fourth generation cephalosporins for food producing animals in the EU in 2017, according to the latest European Surveillance of Veterinary Antimicrobial Consumption (EVSAC) report on the sales of veterinary antimicrobial agents, are summed up in Table 2. Information on sales are calculated as mg of antimicrobial agent per population correction unit (PCU), that is a proxy for the size of the food-producing animal population [94]. Penicillin sales in the EU differed greatly, from 1.6 mg/PCU in Norway, up to 70.3 mg/PCU in Italy and 81.1 mg/PCU in Cyprus. The sales of first- and second-generation cephalosporins were lower than those of penicillins and third- and fourth-generation cephalosporins. No sales of first- and second-generation cephalosporins were reported in Iceland and Norway, while low sales (0.01 mg/PCU or less) were registered in Sweden, Romania and Greece, and the highest sales were in Slovakia (0.4 mg/PCU). The sales of third and fourth generation cephalosporins within the EU were the highest in Estonia (0.8 mg/PCU), followed by Luxembourg and Portugal (both 0.6 mg/PCU), and Czech Republic and Hungary (0.5 mg/PCU) [94]. Some countries, like Belgium, Bulgaria, Denmark, Finland, Iceland, Ireland, The Netherlands, Norway, Sweden and United Kingdom have low sales (0.1 mg/PCU or less) of these antibiotics.

Overall, in sales of penicillins, third and fourth generation cephalosporins for food-producing animals were the lowest in Northern European countries. Both Eastern and Southern European countries have highest sales of third and fourth generation cephalosporins. At the same time, Southern European countries showed the lowest sales of first and second generation cephalosporins. It must be taken into consideration that these data are for all food-producing animals and not only for pigs.

## 5. ESBL Producing *E. coli* in the Pork Production Chain

With the intensive use of antimicrobials in veterinary medicine, the resistance levels against some antibiotics in food-producing animals have increased rapidly since the first reported cases of AMR. Animal farms and slaughterhouse wastewater treatment plants have been shown to contain a more diverse set of plasmids and gene cassettes, compared to hospital wastewater, and they might be considered a hotspot for horizontal ARG transfer [73]. In a study performed in Germany, an assessment of the prevalence of ESBL producing *E. coli* in slaughterhouses and municipal wastewater treatment plants was carried out and it showed that both the wastewaters from slaughterhouses (85.1%) and municipal wastewater treatment plants (97.2%) were highly contaminated with ESBL producing *E. coli* [82]. In fact, the personnel that works at abattoirs slaughtering, dehairing or eviscerating the animals, and the staff in municipal wastewater treatment plants, have a high risk of possible colonization by resistant (also ESBL-producing) bacteria [82]. Another route of transmission at farm level could be through the inhalation of the air or dust in the stables, which can carry ESBL producing bacteria [95].

### 5.1. Prevalence of ESBL Producing E. coli in Fattening Pigs

In 2012 EFSA gathered information on cefotaxime- and ceftazidime-resistant *E. coli* in fattening pigs from seven EU Member States (MS) and one non-MS that reported data, i.e., Denmark, The Netherlands, France, Belgium, Austria, Hungary, Poland, and Switzerland [96]. In 2015 and 2017 EFSA collected information about the prevalence of antimicrobial resistant *E. coli* as indicator bacteria isolated from fattening pigs and pork meat in the frame of the AMR routine monitoring program, according to Commission Implementation Decision 2013/652/EU [19]. In 2015, 28 EU Member States and two non-EU Member States (Norway and Switzerland) reported data on presumptive ESBL producing *E. coli* in fattening pigs [18]. In 2017, 31 countries participated in reporting the data to EFSA, 28 Member States and three other European countries (Norway, Iceland and Switzerland).

Among the participating countries in 2012, the highest prevalence of cefotaxime and ceftazidime resistant *E. coli* was reported in Belgium, 2.9% and 3.4%, respectively, followed by Poland (2.6%). No cefotaxime and ceftazidime resistant *E. coli* was reported in the Netherlands and Austria.

In 2017, the overall prevalence of presumptive ESBL producing *E. coli* in fattening pigs in the EU was 30.62%, that is slightly higher than reported in 2015 (30.2%) (Table 3) [19]. Overall, in Northern European countries, the prevalence of ESBL producing *E. coli* is lower than in other regions (Western, Eastern and Southern Europe). Latvia showed the highest prevalence rate both in 2017 (42.3%) and 2015 (40%) among Northern European countries, while Norway, Finland and Iceland had low prevalence rates (0.7% to 0%) [18,19]. Within Western European countries, in 2017, Belgium had the highest prevalence rate, while Switzerland and The Netherlands the lowest, with a 60.7%, 11.5% and 11% prevalence, respectively. Additionally, in 2015, the highest prevalence of ESBL producing *E. coli* was detected in Belgium (54.7%), and the lowest in Switzerland (17%) and The Netherlands (10.3%). Interestingly, Belgium has the highest sales of penicillins, and first- and second-generation cephalosporins within the Western European countries.

With regards to Eastern European countries, in 2017, Hungary had the highest prevalence rate (56.2%), while in 2015 Bulgaria was the country with the highest one (49.8%). The lowest rates in 2017 were registered in the Czech Republic. In 2015 Slovakia had a prevalence of 9.1%, while in 2017 it escalated to 34%, which was the highest increase in all the EU. In addition, Slovakia had the highest sales of early generation cephalosporins within the EU. Southern Europe showed the highest prevalence of presumptive ESBL producing *E. coli* in fattening pigs, both in 2015 and 2017. Spain had the highest prevalence registered, both in 2015 (81.5%) and in 2017 (80.3%), and Cyprus the lowest, even though it had the highest sales of penicillins in the EU (Table 2 and Table 3).

An increase in the prevalence of ESBL producing *E. coli* in fattening pigs has been reported in five out of eight countries (participating in reporting data since 2012). In The Netherlands, Belgium, Austria, Hungary and Poland, the prevalence of ESBL producing *E. coli* has increased dramatically. For example, in 2012 Austria reported 0% prevalence of cefotaxime- or ceftazidime-resistant *E. coli* isolates from fattening pigs, while in 2015 the prevalence increased to 48.2%, and in 2017 to 58.8%. Similar increases have been reported by Belgium, where resistance to cefotaxime was 2.9% and to ceftazidime 3.4% in 2012, however in 2015 ESBL phenotypes were detected in 54.7% of the *E. coli* isolates from fattening pigs and in 2017 in 60.7%. In Denmark, France and Switzerland, an increase from 2012 to 2015 was detected, but in 2017, the prevalence of ESBL producing *E. coli* decreased again.

In 2010, a voluntary ban on cephalosporin use in Danish pig production was approved in Denmark. After the ban (6–10 month period) the occurrence of extended-spectrum cephalosporinase (ESC)-producing *E. coli* in pigs at slaughterhouse decreased significantly, although ESC producing *E. coli* could still be detected in the herds for a period of time even after the cease of the use of third and fourth generation cephalosporins [97]. Likewise, a prevalence of ESBL-producing *E. coli* of 79% has been reported on farms with high usage of cephalosporins, while on farms with no consumption of these antibiotics the prevalence was 20% [98]. These results indicate the presence of a direct correlation between the consumption of cephalosporins and the prevalence of ESBL-producing *E. coli* in pigs [97,99].

The slaughter process of pigs includes many steps; some of them ensure the decrease of microbial contaminants, but some increase the risk of contamination. Thus, after scalding, singeing, carcass washing and rinsing steps, and chilling, the carcass microbial contamination and the numbers of *Enterobacteriaceae* decrease [100]. After scraping and polishing steps, re-contamination of pig carcasses can happen. Indeed, *E. coli* has been isolated from scraper/dry polisher blades prior to the start of the process [22]. The highest risk of *Enterobacteriaceae* contamination in the slaughterhouse and during meat processing is the evisceration step, as it includes the removal of intestines, which contain high amounts of *E. coli*.

Microbiological criteria aiming at improving the hygiene during slaughtering are set in the EU legislation (Regulation 2703/2005) [101]. According to the EU legislation, hot water can be used to reduce *E. coli* levels on pig’s carcasses. However, other decontamination methods, apart from hot water, can be applied, if they have been previously evaluated and authorized for use [102]. At the same time, process water that accumulates in different phases of the slaughter process might be one of the possible cross-contamination routes during the slaughtering process [82].

### 5.2. Prevalence of ESBL Producing E. coli in Pork Meat

Pork meat is the most widely consumed meat worldwide. It accounts for more than 36% of the meat intake of the world, followed by poultry with a 35% and beef with a 22% [103]. The EU is the world’s second largest pork producer after China, and exports around 13% of the produced meat [104]. The consumption of pork within the EU has increased in the last decade. The main pork meat producers in the EU are Germany, Spain and France and they generate half of the total EU production by number of animals [105].

In 2015, 23 EU Member States, Norway and Switzerland reported data on presumptive ESBL producing *E. coli* in pork meat [18]. The prevalence of ESBL-producing *E. coli* in pork meat was lower than in fattening pigs, and varied from 21.3% in Portugal and 20.8% in Bulgaria down to 0.3% in Sweden and 0% in Norway and Finland. In 2015 the overall prevalence of indicator *E. coli* with ESBL phenotypes in the EU Member states and Norway and Switzerland from pig caecum samples was 30.2%, but from pork meat 6.2%.

In 2017, 28 Member States and three other European countries (Norway, Iceland and Switzerland) reported data on presumptive ESBL producing *E. coli* in pork meat [19]. Prevalence in pork meat (gathered at retail) was lower than in 2015 and varied from 11.1% in Malta down to 0% in Luxembourg, Sweden, Finland, United Kingdom, Iceland and Norway. In 2017 the overall prevalence of indicator *E. coli* with ESBL phenotypes in the EU Member states and Norway, Iceland and Switzerland from pig caecum samples was 30.6%, but from pork meat 4.2% [19].

During a two-year (2015–2017) monitoring study, the prevalence of ESBL-producing *E. coli* in pork meat has decreased. The majority of countries show a decrease on prevalence of ESBL-producing *E. coli* in pork meat, with the largest decrease being reported by Portugal, from 21.3% down to 8.2%. Nevertheless, eight countries reported increases; the highest increase was reported by Croatia, from 1.4% in 2015 to 5.1% in 2017.

As discussed in previous sections, many ESBL enzymes are encoded by genes located in plasmids and can be also found in bacteria from healthy animals, although resistance to some antibiotics such as ceftazidime and cefotaxime is higher among isolates from sick animals [106]. Food-producing animals, like poultry or pigs, carrying ESBL producing *Enterobacteriaceae*, even without showing any clinical signs of disease, are possible reservoirs of ESBLs that can be transferred to humans via the food chain by inappropriate handling and inadequate cooking of meat [71]. The transfer of commensal *E. coli* bacteria from animal intestines to meat can occur during the slaughter process. A study from Schill et al. showed that fresh pork meat can be a source of ESBLs, even though standard microbiological hygiene parameters were satisfactory [25]. ESBL-producing *E. coli* have been reported in meat products since 1990s [107,108]. Generally, the ingestion of ESBL producing species does not result in the colonization or disease of the consumer. However, the exposure to these bacteria may facilitate the dissemination of ESBL genes to the human intestinal microbiome [86,109]. Indeed, it has been described that a plasmid from *E. coli* carrying the *bla*_CTX-M-1_ gene was transferred from pigs to piggery workers [110]. These transfer events pose a risk of colonization and spread of ESBLs in the human population.

Compared to 2015, in 2017 the prevalence of presumptive ESBL-producing *E. coli* in pig’s caecum samples has increased in most EU countries. However, the contamination level in pork meat has decreased. It must be considered that in 2015 six countries were not included in the EFSA’s report on antimicrobial resistance in zoonotic and indicator bacteria. The high prevalence of ESBLs in pigs and the relatively low prevalence in pork meat from official surveillance data demonstrates that animals carry ESBL-producing *E. coli* in their intestinal microbiota, but throughout the slaughter process carcasses are not heavily contaminated with the intestinal content and the ESBL-producing bacteria are somehow removed during the slaughtering and processing steps [19]. It must be taken into account that the prevalence of ESBL-producing *E. coli* in pigs is obtained from screenings performed from the caecum contents, which is a natural habitat of commensal *E. coli,* and not from swabs from the pig skin or other parts of the carcass, and this is likely the reason for the higher prevalence of ESBL-producing *E. coli* in pigs than in pork meat. As well, some countries have a high imported meat capacity, therefore, no direct relationship between the resistance in pig’s caecum and pork meat can be performed [19].

Based on antibiotic sales data in 2017 and presumptive ESBL-producing *E. coli* prevalence in fattening pigs and in pork meat, the correlation level between the usage of β-lactam antibiotics and the resistance status of *E. coli* isolates has been characterized in the literature [111] ( Table 4; Table 5).

**Figure 2:**
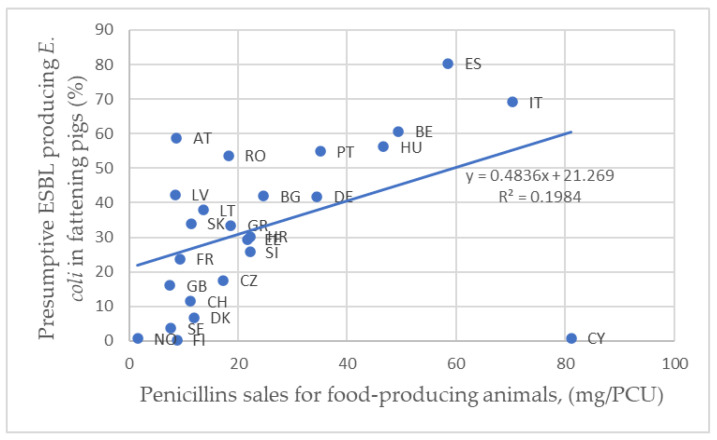
Correlation between presumptive ESBL *E. coli* prevalence in fattening pigs and sales of penicillins in 2017. Country codes used according to ISO 3166.

**Figure 3:**
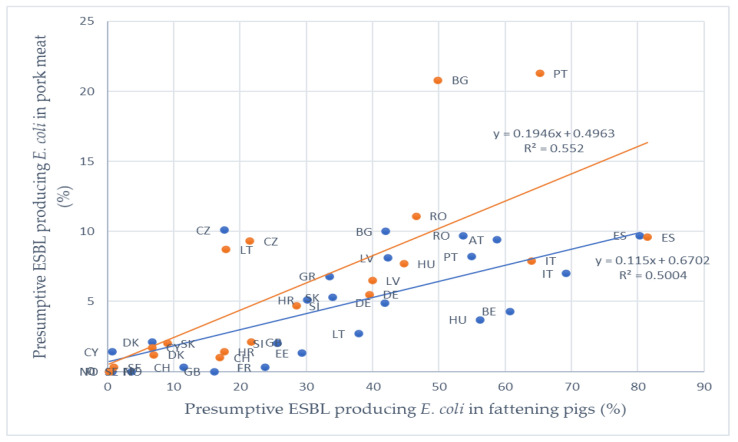
Correlation between the prevalence of presumptive ESBL producing *E. coli* in pork meat and in fattening pigs in 2015 (orange color) and 2017 (blue color). Country codes used according to ISO 3166.

As indicated in Table 4, the correlation between the cephalosporin sales and the prevalence of presumptive ESBL *E. coli* in fattening pigs in 2017 was very low. However, a relevant correlation between sales of penicillins and the prevalence of presumptive ESBL *E. coli* in fattening pigs in 2017 was established (Figure 2). No official data on the sales of antibiotics are available for different food-producing animals, instead they are all summed up, therefore the results should be analyzed with precaution. A strong correlation was observed between the prevalence of presumptive ESBL producing *E. coli* in fattening pigs and in pork meat in 2017 and 2015 (Figure 3). Due to the lack of information on the prevalence of presumptive ESBL producing *E. coli* in fattening pigs or pork meat in 2015, six countries (Ireland, Iceland, Luxembourg, Netherlands, Poland and Malta) were excluded from the analyses.

## 6. Literature Review on the Prevalence of ESBL Producing *E. coli* in Pork Meat

A literature search was conducted with the database Scopus using the search string (ESBL OR “extended spectrum β lactamas*”) AND (occurrence OR prevalence) AND (Escherichia OR coli OR “E. coli”) AND (pork OR “ground pork” OR porcine OR “minced pork”). The literature search was limited to research articles published from 2000 to 2020 in the EU. The literature search was conducted until June 2020. In total, 902 articles were retrieved. A first screening was undertaken by revising titles and abstracts to remove articles not related to the topic. Then, for those articles which progressed to the next steps, the full text was analyzed to determine whether the inclusion criteria were met. Articles lacking information on the origin of the meat, the meat type, the number of screened samples, or the number/prevalence of positive isolates were discarded. At last, only 14 studies complied with the purpose of the review or were available. They are summarized in Table 6. The main purpose was to extract information on the prevalence of different ESBL producing *E. coli* in pork meat. However, different approaches were applied in these studies to determine the resistance status, e.g., isolate antimicrobial susceptibility determined by micro-broth dilution method using media supplemented with cefotaxime or ceftazidime, double synergy differential test, and sometimes confirmation by polymerase chain reaction (PCR) and DNA sequencing analyses. These studies examined both fresh and frozen pork meat from various parts of the carcass, with ground pork being also examined in some studies. Samples were taken from different meat production/retail places, such as slaughterhouses, supermarkets, butcher shops, meat-packaging, and meat-processing companies. Prevalence of ESBL producing E. coli varied from 0% up to 25%. However, multiple factors must be considered, like the sample size, detection method used, and meat origin, therefore, the collected data should be analyzed with precaution. Interestingly, a large variability existed even among studies conducted in the same country. Thus, for instance, in Germany alone the prevalence rates largely varied, from 0.7% up to 17.5% [25,112].

In eight out of fourteen studies, ESBL coding genes were determined by PCR analysis. In all studies apart from the one conducted in the Czech Republic [113], CTX-M genes dominated. These findings agree with those of other studies before indicating that, nowadays, CTX-M is the most prevalent ESBL in livestock [4,17]. TEM β-lactamases were the second most prevalent type identified, with the highest rate (40.6%) being detected in pork meat originating from the Czech Republic. SHV genes were present only in five studies with a relatively low prevalence rate, ranging from 0.2 to 3.85%.

## 7. Conclusions

The prevalence of ESBL producing *E. coli* in the EU varies significantly among countries, both in fattening pigs (swine caecum samples) and pork meat. Different approaches regarding the use of antibiotics are applied in the EU. For instance, a voluntary ban on cephalosporins was adopted in Denmark in 2010 and since then the levels of ESBL producing *E. coli* isolated from pig caecum content have decreased. Overall, the prevalence of ESBL producing *E. coli* isolated from pigs is increasing over the years. The prevalence of presumptive ESBL producing *E. coli* in pork meat in the EU varies a lot, and even in countries where the prevalence is low or close to zero, it is possible that meat imported in the country from inside or outside the EU containing high levels of ESBL producing *E. coli* may pose a risk for the consumer. Some strategies to deal with the global AMR problem have been globally discussed and will in principle be also relevant in the fight against the spread of ESBL producing bacteria along the pork production chain. For example, good governance and usage principles of antimicrobials, monitoring of antimicrobial usage and resistance, and prevention measures, including biosecurity, should be implemented or revised to reduce the risk of introducing or spreading AMR bacteria into a herd or at farm level.

The personnel at swine industries (at farm level and slaughterhouses) are exposed to ESBL producing *E. coli*, which poses a risk of possible intestinal colonization with AMR pathogenic bacteria. Pork meat could be cross-contaminated during the slaughter process and by the personnel. Overall, the information available in the literature shows the urgent need to reconsider the responsible use of antimicrobials at farm level. Although the prevalence of ESBL producing *E. coli* is lower in pork meat than in pig caecum samples in most of the EU countries, pork meat can act as a transmission route for this group of critically important AMR bacteria. As pork meat can be cooked medium done and some pork products are not cooked, but fermented, it poses a risk of transferring the ESBL producing *E. coli* to the human gut microbiome upon consumption of contaminated meat. As *E. coli* is not a heat-resistant bacterium, it does not survive intense heat treatments. Therefore, appropriate cooking temperatures and times should be applied while preparing pork meat. Both in the pork production industry and in households, good hygiene practise should be followed.

## Figures and Tables

**Table 1:** Classification of TEM, SHV and CTX-M β-lactamases. Adapted from [27].

β-Lactamase	Molecular Weight of the Enzyme	Bush-Jacoby Medeiros Class	Ambler Class	Active Site	Preferential Substrate	Gene	Organisms
TEM	~29,000 Da	2be	A	Serine	Penicillins and 1st–4th generation cephalosporins	Pl*	*Enterobacteriaceae, Haemophilus influenzae, Neisseria gonorrhoeae, Pseudomonas aeruginosa*
SHV							*Enterobacteriaceae*
CTX-M							*Enterobacteriaceae*

*Pl—plasmid mediated.

**Table 2:** Penicillin and cephalosporin sales in the EU for food-producing animals, in mg per population correction unit (mg/PCU) in 2017 [94].

Region	Country	Penicillin Sales for Food-Producing Animals, (mg/PCU) in 2017	1st- and 2nd-gen. Cephalosporin Sales for Food-Producing Animals, (mg/PCU) in 2017	3rd- and 4th-gen. Cephalosporin Sales for Food-Producing Animals, (mg/PCU) in 2017
Northern Europe	United Kingdom	7.5	0.1	0.1
	Sweden	7.7	<0.01	<0.01
	Finland	8.8	0.03	<0.01
	Latvia	8.5	0.2	0.3
	Lithuania	13.6	0.1	0.2
	Ireland	9.7	0.3	0.1
	Estonia	21.7	0.2	0.8
	Denmark	11.9	0.02	<0.01
	Iceland	3.3	0	<0.01
	Norway	1.6	0	<0.01
Western Europe	Luxembourg	6.8	0.1	0.6
	Netherlands	12.6	0.04	<0.01
	France	9.3	0.2	0.02
	Germany	34.5	0.1	0.4
	Belgium	49.4	0.2	0.1
	Austria	8.6	0.05	0.2
	Switzerland	11.2	0.1	0.2
Eastern Europe	Bulgaria	24.7	0.02	0.1
	Czech Republic	17.3	0.3	0.5
	Croatia	22.3	0.2	0.2
	Hungary	46.7	0.1	0.5
	Poland	54.1	0.1	0.2
	Romania	18.2	<0.01	0.2
	Slovakia	11.4	0.4	0.4
Southern Europe	Cyprus	81.1	0.03	0.4
	Spain	58.5	0.03	0.2
	Greece	18.6	<0.01	0.1
	Italy	70.3	0.2	0.4
	Malta	12.1	0.1	0.2
	Portugal	35.1	0.1	0.6
	Slovenia	22.2	0.1	0.2
	Total	23.2	0.1	0.3

**Table 3:** Prevalence of ESBL-producing, cefotaxime- and ceftazidime-resistant, *E. coli* isolates from fattening pigs and pork meat in the EU, 2012–2017. Adapted from [18,19,96].

Region	Country	Resistance to Cefotaxime in *E. coli* Isolates from Pigs % (2012)	Resistance to Ceftazidime in *E. coli* Isolates from Pigs % (2012)	Presumptive ESBL *E. coli* Prevalence in Fattening Pigs % (2015)	Presumptive ESBL *E. coli* Prevalence in Pork Meat % (2015)	Presumptive ESBL *E. coli* Prevalence in Fattening Pigs % (2017)	Presumptive ESBL *E. coli* Prevalence in Pork Meat % (2017)
Northern Europe	United Kingdom	-	-	21.7	2.1	16.1	0
	Sweden	-	-	1	0.3	3.7	0
	Finland	-	-	0.3	0	0.3	0
	Latvia	-	-	40	6.5	42.3	8.1
	Lithuania	-	-	17.9	8.7	37.9	2.7
	Ireland	-	-	10.3	-	18.5	2.3
	Estonia	-	-	29.9	2.7	29.4	1.3
	Denmark	0.7	-	7	1.2	6.8	2.1
	Iceland	-	-	-	-	0	0
	Norway	-	-	0.4	0	0.7	0
Western Europe	Luxembourg	-	-	48.3	-	38.1	0
	Netherlands	0	0	10.3	-	11	0.7
	France	2	2	34.7	1.5	23.8	0.3
	Germany	-	-	39.5	5.5	41.8	4.9
	Belgium	2.9	3.4	54.7	11.8	60.7	4.3
	Austria	0	0	48.2	9.3	58.8	9.4
	Switzerland	1.1	1.1	17	1	11.5	0.3
Eastern Europe	Bulgaria	-	-	49.8	20.8	42	10
	Czech Republic	-	-	21.5	9.3	17.6	10.1
	Croatia	-	-	17.6	1.4	30.2	5.1
	Hungary	1.5	-	44.7	7.7	56.2	3.7
	Poland	2.6	2.6	26.8	-	31.9	3.7
	Romania	-	-	46.6	11.1	53.7	9.7
	Slovakia	-	-	9.1	2	34	5.3
Southern Europe	Cyprus	-	-	6.8	1.7	0.8	1.4
	Spain	-	-	81.5	9.6	80.3	9.7
	Greece	-	-	32.8	5.8	33.5	6.8
	Italy	-	-	64^a^	7.9^a^	69.2	7
	Malta	-	-	-	-	17.9	11.1
	Portugal	-	-	65.2	21.3	54.9	8.2
	Slovenia	-	-	28.5	4.7	25.7	2
	Total	1.4	1.5	30.2	6.2	30.6	4.2

^a^—results reported from molecular analyses; - no data available.

**Table 4:** Correlation between antibiotic sales and prevalence of presumptive ESBL-producing *E. coli* in fattening pigs, 2017.

Antibiotic Sales		Prevalence	Correlation Coefficient	Correlation Level [111]
Penicillin sales for food-producing animals, (mg/PCU)	&	Presumptive ESBL *E. coli* prevalence in fattening pigs	0.464	Middle
1st and 2nd generation cephalosporin sales for food-producing animals, (mg/PCU)	&	Presumptive ESBL *E. coli* prevalence in fattening pigs	0.180	Very low
3rd and 4th generation cephalosporin sales for food-producing animals, (mg/PCU)	&	Presumptive ESBL *E. coli* prevalence in fattening pigs	0.074	Very low

**Table 5:** Correlation between prevalence of presumptive ESBL *E. coli* in fattening pigs and pork meat.

Prevalence in Fattening Pigs		Prevalence in Pork Meat	Correlation Coefficient	Correlation Level [111]
Presumptive ESBL *E. coli* prevalence in fattening pigs in 2017	&	Presumptive ESBL *E. coli* prevalence in pork meat in 2017	0.626	Strong
Presumptive ESBL *E. coli* prevalence in fattening pigs in 2015	&	Presumptive ESBL *E. coli* prevalence in pork meat in 2015	0.735	Strong

**Table 6:** Prevalence of ESBL producing *E. coli* in pork meat, obtained from studies published in the EU.

Meat Origin	Sampling Period	Type of Meat	Meat Origin	Number of Samples	Prevalence of ESBL Producing *E. coli* (%)	ESBL Encoding Genes			Reference
						CTX-M (%)	TEM (%)	SHV (%)	
Italy	2016–2017	Carcasses	Slaughterhouse	200	10	11.5	3.5	0.5	[114]
		Sausages, meat slices, loin, salami dough, cotechino, thighs for ham production	Supermarkets	446	2	1.8	1.4	0.2	
Belgium	2015–2016	Head	Slaughterhouse	104	25	-	-	-	[115]
		Belly	Slaughterhouse	104	7	-	-	-	
		Ham	Slaughterhouse	103	3	-	-	-	
		Loin	Slaughterhouse	104	1	-	-	-	
Germany	2014	Carcasses	Meat processing company	63	17.5	15.9	4.8	0	[25]
Italy	2013–2014	Ground pork	Food market	200	0	-	-	-	[116]
England, Wales, and Scotland	2013–2014	Pork	Supermarkets, discount store, convenience stores, butchers	79	3	2	0	0	[117]
Switzerland	2013	Pork	Meat-packaging plant	50	0	-	-	-	[118]
Germany	2012–2013	Pork meat	Butcher shops, supermarkets, farmer’s markets, direct marketer, restaurants, canteens	282	12.1	11.3	0.7	0.4	[119]
		Ground pork		214	13.6	10.7	0.9	0.9	
Poland	2012–2013	Pork	Slaughterhouse	78	5	5.13	0	3.85	[120]
Czech Republic	2012–2013	Pork	Supermarkets	110	3.1	0	40.6	0	[113]
Denmark	2010–2011	Frozen or fresh pork	Retail stores, outlets	44	2	2.3	2.3	0	[121]
Germany				44	7	6.8	4.5	0	
Italy				20	15	10	10	0	
Finland, The Netherlands, Poland, Spain				31	9.6	0	0	0	
Denmark	2009	Frozen or fresh pork	Retail store, outlets	153	2	1.31	0	0	[112]
Germany^a^				142	0.7	-	-	-	
The Netherlands				16	0	-	-	-	
Austria	2009	Ground pork	Supermarkets	27	3.7	-	-	-	[122]
Iceland	2006–2007	Pork	Meat-processing plants	60	0	-	-	-	[123]
Spain	2006–2007	Pork	Supermarket	12	25	-	-	-	[124]

^a^ no information on the gene/s present in the isolate; - not tested.
